# How might acupuncture work? A systematic review of physiologic rationales from clinical trials

**DOI:** 10.1186/1472-6882-6-25

**Published:** 2006-07-07

**Authors:** Howard H Moffet

**Affiliations:** 1 Kaiser Permanente – Division of Research, Oakland, CA, USA

## Abstract

**Background:**

Scientific interest in acupuncture has led numerous investigators to conduct clinical trials to test the efficacy of acupuncture for various conditions, but the mechanisms underlying acupuncture are poorly understood.

**Methods:**

The author conducted a PubMed search to obtain a fair sample of acupuncture clinical trials published in English in 2005. Each article was reviewed for a physiologic rationale, as well as study objectives and outcomes, experimental and control interventions, country of origin, funding sources and journal type.

**Results:**

Seventy-nine acupuncture clinical trials were identified. Twenty-six studies (33%) offered no physiologic rationale. Fifty-three studies (67%) posited a physiologic basis for acupuncture: 33 (62% of 53) proposed neurochemical mechanisms, 2 (4%) segmental nervous system effects, 6 (11%) autonomic nervous system regulation, 3 (6%) local effects, 5 (9%) effects on brain function and 5 (9%) other effects. No rationale was proposed for stroke; otherwise having a rationale was not associated with objective, positive or negative findings, means of intervention, country of origin, funding source or journal type. The dominant explanation for how acupuncture might work involves neurochemical responses and is not reported to be dependent on treatment objective, specific points, means or method of stimulation.

**Conclusion:**

Many acupuncture trials fail to offer a meaningful rationale, but proposing a rationale can help investigators to develop and test a causal hypothesis, choose an appropriate control and rule out placebo effects. Acupuncture may stimulate self-regulatory processes independent of the treatment objective, points, means or methods used; this would account for acupuncture's reported benefits in so many disparate pathologic conditions.

## Background

Clinical trials often test a causal association between intervention and outcome [1]. However, Ernst has asserted that, "Viewed from a scientific perspective, acupuncture is rarely, if ever, a causal therapy" [2]. Perhaps acupuncture is not causal – no more so than flipping a light switch "causes" illumination. However, if acupuncture is a causal intervention, investigators should be able to suggest a biological pathway, hypothesis or rationale for how it might work.

"How might acupuncture work?" is asked and answered on the website of the National Center for Complementary and Alternative Medicine (NCCAM) of the National Institutes of Health: "It is proposed that acupuncture produces its effects through regulating the nervous system, thus aiding the activity of pain-killing biochemicals such as endorphins and immune system cells at specific sites in the body. In addition, studies have shown that acupuncture may alter brain chemistry by changing the release of neurotransmitters and neurohormones and, thus, affecting the parts of the central nervous system related to sensation and involuntary body functions, such as immune reactions and processes that regulate a person's blood pressure, blood flow, and body temperature"[3]. More detailed rationales for acupuncture are readily available [4].

The CONSORT (Consolidated Standards of Reporting Trials) Statement recommends that clinical trial authors "suggest a plausible explanation for how the intervention under investigation might work" [5]. Clinical trials of acupuncture might be more useful if they had not just a hypothesis about efficacy, but also a hypothesis about a mechanism. The purpose of this study was to determine to what extent contemporary acupuncture clinical trials proposed physiologic rationales and present the findings in the context of other characteristics of the studies.

## Methods

The author sought a fair sample of acupuncture clinical trials and conducted a PubMed search using the keyword "acupuncture," further limiting the search to "clinical trials" published in "English" in "2005." The author obtained and reviewed a copy of every article identified from this search; articles were excluded if they were not actually clinical trials of acupuncture; letters and brief articles were also excluded as they were unlikely to have the details sought. Each article was reviewed for a physiologic rationale; that is, a description of any human physiologic process as an explanation linking the intervention to the outcome. Articles were also reviewed for their objectives (indications or experimental conditions) and outcomes of interest, the experimental and control interventions, country of origin, funding sources and type of journal. Rationales were not counted if based only on acupuncture theory or practice or on published or historical reports; a description of some physiologic process was required. This study had no external funding.

## Results

**Figure 1:**
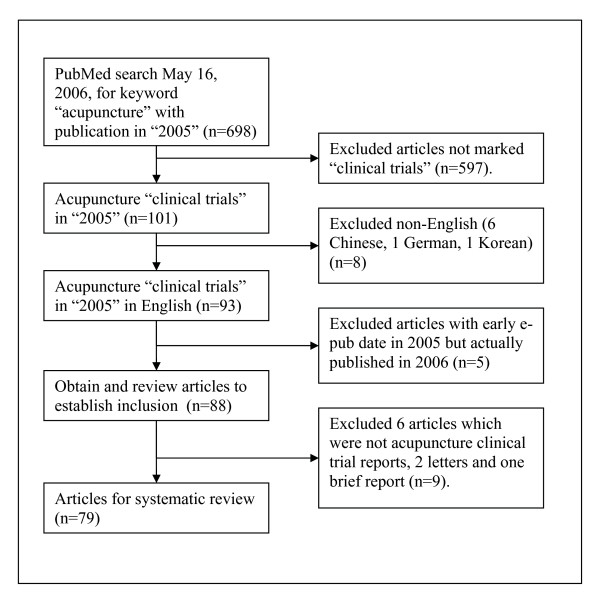
Flow chart for selection of clinical trials of acupuncture.

The PubMed search on May 16, 2006, using the keyword "acupuncture" yielded 698 publications in 2005; 101 were indexed as acupuncture clinical trial reports, of which 93 were published in English. Five articles had an advance e-publish date in 2005 but were formally published in 2006 and were excluded. Six articles were excluded because they were not, in fact, clinical trial reports; also, two letters and one brief article were excluded. The study sample includes all remaining articles (n = 79) [6-84]. (Figure 1) After reviewing these papers, they were categorized according to their rationale: none, neurochemical, segmental ("gate control"), autonomic regulation, local effects, functional effects in the brain or other effects.

### Rationales

Twenty-six articles (33%) offered no discernible physiologic rationale for how acupuncture might work (Table 1). Indications with no rationale include addiction [42], auditory hallucinosis [35], breech presentation [13], chronic fatigue syndrome [49], chronic sinusitis [70], depression [67], irritable bowel syndrome [29], mental fatigue [32], overactive bladder syndrome [27] and stroke rehabilitation [62,80,82].

Fifty-three articles (67%) proposed some physiologic process or mechanism attributing the effects of acupuncture to neurochemical, segmental ("gate theory"), autonomic regulation, local effects, effects on brain function or other effects. Most rationales were less specific than the NCCAM website, but at least suggested a non-metaphysical explanation, e.g., "Acupuncture functions by regulating the physiological state of the human body" [66]. Some explanations were inadequate, such as this for treating Parkinson's disease: "needles promote the release of endorphins and improve local blood flow" [20]. The best rationales were informative, albeit brief, for example, that for analgesia, "acupuncture stimuli act as a central nervous system input that can activate the descending antinociceptive pathway to release endogenous opioids to deactivate the ascending nociceptive pain pathway" [31]. Except for studies which measured specific physiologic outcomes, few studies directly tested their rationale. Studies which cited multiple rationales were assigned to the one which appeared to be primary.

The dominant rationale cited by 33 studies is that acupuncture stimulates the release of neurochemicals (usually endogenous opioids [beta endorphins, enkephalins and dynorphins] or serotonin). Among 36 studies of analgesia, this rationale was cited by 20 (56% of 36) articles [9,10,15,16,18,21,24-26,30,31,37,40,43,52-54,65,68,72]. This rationale was also used to explain the effects of acupuncture on nausea/vomiting [6,11,41,55], obesity [12], Parkinson's [20], irritable bowel [29], immune function [45], lower esophageal sphincter relaxations [84], blood pressure [71], post-menopausal vasomotor symptoms [56], colitis [83] and sleep quality [22].

Two studies identified segmental effects or "gate theory" as a primary mechanism specifically for analgesia [17,47], though five others referred secondarily to this rationale [15,24,26,30,54]. Sensory input from acupuncture is thought to block or interfere with nociceptive pain signals at a spinal level.

Six studies referred to modulatory effects of acupuncture on the autonomic nervous system [14,36,48,50,58,73]. However, several other studies that did not refer to autonomic regulation in their rationale did nonetheless measure outcomes which may reflect autonomic regulation: heart rate [21,45,48,66,82], heart rate variability [14,36,48], blood pressure [21,45,66,71,82], post-menopausal vasomotor symptoms [56] or respiration [82]. Other studies measured outcomes which also suggest possible ANS regulation: effects on smooth muscle [50], sleep quality [15,22,34], urinary continence [27], sweat rate [58], rate of transient lower esophageal sphincter relaxations [84] or nausea/vomiting [73].

Three studies referred to local effects of acupuncture on tissues or nerves [61] or mechanical effects on connective tissue [7,44]; other studies secondarily referred to changes in circulation [20,52,54], especially vasodilation [72], or effects on immune function[52,83].

Five studies proposed that acupuncture can have specific functional effects in the brain and used fMRI to correlate acupuncture points to specific areas of the brain with specific sensory or motor functions [38,39,63,74,78]. These raise fascinating possibilities even if no clear mechanism is posited.

Five studies suggested other rationales: that acupuncture promotes homeostasis [66], regulates brain function [59,75], affects sperm motility [64] or suggests that response to acupuncture may vary by patient genotype[60].

### Objectives and outcomes

Studies used appropriate biomedical diagnostic inclusion criteria and outcome measures, although one study did rely on a traditional Chinese medicine (TCM) differential diagnosis to assign subjects to a treatment group [49]. No rationale was proposed for stroke; otherwise, having a rationale was not associated with treatment objective. Sixty-one out of 79 (77%) articles had positive findings, of which 42 (70% of 61) had a rationale; having a rationale was not associated with obtaining positive findings. However, there were positive findings in 17 of 18 studies (94%) whose outcome of interest measured specific physiologic changes (brain blood flow using fMRI imaging [38,39,63,78], muscle blood flow [72], morphologic changes in sperm structure [64], association of acupuncture response to genotype [60], effect on leukocyte circulation [45], heart rate or heart rate variability [14,36,48], blood pressure [66,71], sweat rate [58], tissue impedance [7], sensations of skin piercing [61], post-menopausal vasomotor symptoms [56] or ultrasound imaging of tissue morphology [44].)

### Interventions and controls

These studies utilized a variety of interventions which satisfy a textbook definition of acupuncture as "stimulation of points and channels" [85]. Sixty-four studies used some form of "puncture." Forty-seven studies used acupuncture needles [9,10,15,18-24,26,27,29,31,33,34,36-40,43-46,48,49,51-54,57,59-66,70,72,73,75-77,80,81,83]; others specified the use of electro-acupuncture [7,12,14,16,30,35,47,50,56,58,68], auricular acupuncture [42,79], plum blossom needling [28,83], or blood-letting acupuncture [82]. Fourteen studies used alternatives to needles: transcutaneous electrical stimulation [34,41,71], acupressure [8,32], toothpicks [78], small seeds [49] or wrist bands [69]; others used stimulation by low-power laser [11,25,67,74], topical ointments [6,55], or moxibustion [13]. Having a rationale was not associated with the means of intervention.

There were a variety of control procedures: "placebo" needles which do not puncture the skin, "sham" acupuncture which does puncture the skin (but at alternate points or non-points, near or far from true points), transcutaneous electrical stimulation or laser devices with the power "off", placebo ointment, usual care or alternative treatments.

Acupuncture point selection was usually based on traditional indications or functions (e.g., to balance the yin and yang). No study proposed in its rationale that the neurochemical effects of acupuncture are dependent on point selection nor were important distinctions in physiologic effects made among the various means (needles, pressure, electricity, laser, heat or ointment) or methods (depth, style, frequency or intensity) of stimulation, except for differences in sensations experienced by subjects.

### Country, funding, journal type

The studies originated in 21 countries and were published in 52 different journals. No funding sources were reported in 34 studies (43%); major funding was provided by U.S. National Institutes of Health [16,19,44,76], especially NCCAM [7,9,10,32,33,38,43,47] and from German national insurance providers [46,52,54,81]. NIH-funded trials were no more likely to have a rationale than other studies. Seventeen studies were published in three journals of acupuncture or Chinese medicine [12,14,22,25,28,34-36,39,49-51,61,67,75,82,83] and eight were published in 2 journals of complementary/alternative medicine [7,32,33,41,47,60,70,73]; the remaining 54 were published in 47 medical journals. Having a rationale was not associated with country, funding source or journal type.

## Discussion

Seventy-nine acupuncture clinical trials reports were reviewed and 53 (67%) had some rationale for the use of acupuncture. The study interventions stimulated points using needles, electricity, lasers, pressure, heat or ointments compared to various controls. No rationale was proposed for stroke; otherwise, having a rationale was not associated with objective, positive findings, means of intervention, country of origin, funding source or journal type. The dominant rationale involved release of neurochemicals (usually endogenous opioids [beta endorphins, enkephalins and dynorphins] or serotonin).

No study proposed that the neurochemical effects of acupuncture depend on point selection. No study claimed to select points based on neurochemical effects. However, it should be noted that the locations of traditional points are well-established and often correspond to underlying nerves; thus, the selection of traditional points over "non-points" may be justified. Also, the local and segmental effects would logically depend on the needling sites. Certainly, no study proposed that the neurochemical effects depend on means (needles, pressure, electricity, laser, heat or ointment) or method (depth, style, frequency or intensity) of stimulation. While there is great emphasis on point selection and stimulation technique in traditional acupuncture, the neurochemical response to acupuncture may not depend on them.

The neurochemical rationale was proposed not just for analgesia, but insomnia, nausea/vomiting, obesity, Parkinson's and effects on blood pressure, immune function, colitis, vasomotor symptoms and lower esophageal sphincter relaxation. Further, it cannot be ruled out that other outcomes (e.g., autonomic regulation) may be secondary to neurochemical effects. Also, the same points may be stimulated for many different indications; alternatively, one indication may be treated with disparate point selections, not just between studies, but within studies, even varying by each treatment visit. This suggests that acupuncture simply stimulates self-regulatory processes and would account for acupuncture's reported benefits in so many disparate pathologic conditions.

Hypothesizing a mechanism can aid in selecting an appropriate control intervention. The sham needling with puncture may not be different *in effect *from "true" acupuncture and even the placebo needling is problematic: "Despite no skin penetration, the [placebo needle] tip exerted a mechanical stimulation... [which] may also excite nociceptive primary afferents. No ideal method of placebo stimulation acupuncture exists at present"[48]. In addition, placebos are also associated with neurochemical effects [24,39]. One study concluded that effects of acupuncture may well be "attributable to other mechanisms than perforation of subcutaneous tissue. Repetitive relaxation and being cared for may be just as important" [53]. In an experiment or clinical trial, the control intervention should depend on (i.e., "control for") the hypothesized mechanism and also control for so-called placebo effects. Without a theoretical mechanism and in the absence of a truly inert placebo, it can be difficult to define an appropriate control.

Finally, why is a rationale important? It is not enough to lament that "the mechanisms underlying acupuncture are still poorly understood" [80]. Understanding the physiologic basis of acupuncture may be critical to producing reliable (i.e., reproducible) results. A rationale should be offered as an explanation for the trial intervention, especially when the intervention is poorly understood. The rationale should offer a scientific hypothesis, either explanatory or pragmatic [5], to be tested explicitly or implicitly. Of course, pragmatic trials may proceed without a clear hypothesis about the mechanism involved. Ultimately, however, the effects of acupuncture must be mediated through human physiology and investigators should be able to suggest some possible mechanism(s). Proposing and testing ideas about the underlying mechanisms of acupuncture could eventually lead to a real understanding about how acupuncture does work.

### Limitations

First, only articles published in English were included; six articles in Chinese were excluded [86-91] and one article in Korean was excluded [92]; but studies from Asia were well-represented: China [28,35,48-50,75,82,83], Taiwan [14,15,36], Hong Kong [17], Japan [58,78] and Korea[18,39]. Also, articles indexed in PubMed after May 16, 2006, were not included. Second, the reviewed studies were coded as "having" or "not having" a rationale, but many provided inadequate explanations linking intervention to outcome and no conclusions may be drawn as to whether any of these rationales are valid or relevant to their objectives. Also, while there were no apparent associations between having a rationale and most other characteristics (objectives, interventions, etc), no formal statistical tests were performed to confirm this. It is possible, though unlikely, that there are significant statistical associations which went unnoticed.

## Conclusion

Every clinical trial should attempt to explain the use of the intervention, but many acupuncture trials fail to offer any meaningful rationale. The dominant rationale for acupuncture involves neurochemical responses which appear to be independent of objective, point selection or the means or method of stimulation; this raises questions about the beliefs underpinning this intervention and deserves further investigation. Many studies have attempted to test traditional acupuncture without a physiologic rationale, but proposing a hypothesis for how acupuncture might work is good science and costs nothing; a rationale can help investigators to develop a causal hypothesis, choose an appropriate control and rule out placebo effects. Reviewers making decisions about funding or publication of acupuncture research should seek a physiologic hypothesis from investigators.

## Competing interests

The author(s) declare that they have no competing interests.

## Authors' contributions

The author conceived and designed the study; acquired, analyzed and interpreted the data; and wrote the manuscript.

**Table 1:** Rationales in clinical trials of acupuncture

	Proportion having a rationale
	
All (N = 79)	67%
Objectives	
Analgesia (n = 36)	69%
Nausea/vomiting (n = 8)	62%
Stroke (n = 3)	0%
Other (n = 32)	72%
Findings (positive) (n = 61)	69%
Intervention	
Needles (n = 64)	67%
Other (n = 15)	67%
Country	
Asian (n = 19)	74%
Non-Asian (n = 60)	65%
Funding source	
None reported (n = 34)	65%
NIH (n = 11)	64%
German insurance (n = 4)	50%
Other (n = 30)	73%
Journal type	
Acupuncture (n = 17)	59%
CAM (n = 8)	62%
Medical (n = 54)	70%

## Pre-publication history

The pre-publication history for this paper can be accessed here:


